# Therapeutic Potential of Cell Penetrating Peptides (CPPs) and Cationic Polymers for Chronic Hepatitis B

**DOI:** 10.3390/ijms161226094

**Published:** 2015-11-27

**Authors:** Bénédicte Ndeboko, Guy Joseph Lemamy, Peter. E Nielsen, Lucyna Cova

**Affiliations:** 1 Institut National de la Sante et Recherche Medicale (INSERM) U1052, Cancer Research Center of Lyon (CRCL), Lyon 69003, France; ndeboko@yahoo.fr; 2 Département de Biologie Cellulaire and Moléculaire-Génétique, Faculté de Médecine, Université des Sciences de la Santé, Libreville 241, Gabon; guylemamy@yahoo.fr; 3 Department of Cellular and Molecular Medicine, Departement of Drug Design and Pharmacology, Faculty of Health and Medical Sciences, the Panum Institute, University of Copenhagen, Copenhagen DK 2200N, Denmark; ptrn@sund.ku.dk

**Keywords:** hepatitis B, antiviral therapy, cell penetrating peptides (CPPs), chitosan (CS), catonic polymers, peptide nucleic acids (PNAs), hepatitis B virus (HBV), duck hepatitis B virus (DHBV), DNA vaccine, gene delivery, antigen (Ag)

## Abstract

Chronic hepatitis B virus (HBV) infection remains a major health problem worldwide. Because current anti-HBV treatments are only virostatic, there is an urgent need for development of alternative antiviral approaches. In this context, cell-penetrating peptides (CPPs) and cationic polymers, such as chitosan (CS), appear of particular interest as nonviral vectors due to their capacity to facilitate cellular delivery of bioactive cargoes including peptide nucleic acids (PNAs) or DNA vaccines. We have investigated the ability of a PNA conjugated to different CPPs to inhibit the replication of duck hepatitis B virus (DHBV), a reference model for human HBV infection. The *in vivo* administration of PNA-CPP conjugates to neonatal ducklings showed that they reached the liver and inhibited DHBV replication. Interestingly, our results indicated also that a modified CPP (CatLip) alone, in the absence of its PNA cargo, was able to drastically inhibit late stages of DHBV replication. In the mouse model, conjugation of HBV DNA vaccine to modified CS (Man-CS-Phe) improved cellular and humoral responses to plasmid-encoded antigen. Moreover, other systems for gene delivery were investigated including CPP-modified CS and cationic nanoparticles. The results showed that these nonviral vectors considerably increased plasmid DNA uptake and expression. Collectively promising results obtained in preclinical studies suggest the usefulness of these safe delivery systems for the development of novel therapeutics against chronic hepatitis B.

## 1. Introduction

Chronic hepatitis B virus (HBV) infection remains one of the most serious health problems worldwide, despite the availability of a prophylactic vaccine. Indeed, there are more than 350 million chronic HBV carriers in the world [1] and 15% to 40% of them are expected to progress to serious liver disease such as cirrhosis and hepatocellular carcinoma (HCC) [2].

Current therapeutic strategies approved for chronic hepatitis B treatment can be divided into two groups according to their mechanism of action; immunomodulation agents such as standard and pegylated interferon-α (IFN-α) and oral nucleoside analogues (NUCs) including lamivudine (LAM), telbivudine (LdT), entecavir (ETV), adefovir dipivoxil (ADV) and tenofovir dipivoxil fumarate (TDF) [3].

These antiviral treatments are only virostatic, since they are unable to completely eliminate the covalently closed circular viral DNA (cccDNA), a viral minichromosome that is responsible for the rebound of viral replication after therapy cessation. Thus, development of alternative therapeutic approaches for chronic HBV infection is urgently needed [4].

In this regard, cell-penetrating peptides (CPPs), also known as protein transduction domains (PTDs) or membrane translocation sequences (MTSs) represent one of the most promising molecular mechanisms for passive delivery of biologically active molecules into the cells [5]. These molecules are short cationic or amphipatic peptide sequences of 5–40 amino acids that can traverse mammalian plasma membranes [6,7,8,9].

More than two decades ago, short peptides from HIV’s transactivator of transcription (TAT) protein were first discovered to penetrate the cell membrane and efficiently internalize into the cells [10,11]. The discovery of TAT was quickly followed by the identification of other peptides that exhibited similar behavior, such as Antennapedia, a transcription factor from drosophila known as penetratin (pAntp) [12,13] and VP22, a herpes virus protein [14]. Their ability to translocate across the plasma membranes is confined to short sequences of less than 20 amino acids, which are highly rich in basic residues within proteins. These peptides have been used for intracellular delivery of various cargoes with molecular weighs several times greater than their own [15].

Today, this class has grown to include synthetic CPPs such as polyarginine [16,17,18,19] and polylysine [20,21]. These CPPs are able to translocation various bioactive cargoes into the cells, including both low and high molecular weight molecules such as peptide nucleic acids (PNAs) and DNA vaccines. Cellular delivery using CPPs has several advantages over conventional techniques because it is efficient for a range of cell types and has therapeutic potential [22].

PNA, a third generation antisense agent, represent a promising platform for developing inhibitors of HBV replication. PNA is a synthetic DNA mimic in which the natural nucleic acid backbone is replaced by an uncharged pseudo-peptide backbone consisting of *N*-(2-aminoethyl)-glycine units linked to the nucleic bases [23]. PNAs have attractive potential as possible antiviral agents, due to high binding affinity and specificity for sequence complementary nucleic acid targets and exhibit remarkable stability against enzymatic degradation [24,25,26,27]. We have initially demonstrated that antisense PNA can inhibit DHBV reverse transcription in rabbit reticulocyte lysate system [28]. However, the use of PNAs for antiviral therapy has been hampered by their poor cellular uptake and limited *in vivo* bioavailability. In this regard, conjugation of PNAs to the CPPs considerably enhanced cellular penetration of PNAs. Thus, antisense PNA-CPP conjugates are able to block viral replication *in vitro* and *in vivo* in experimental models of HBV infection, as documented by us in a preclinical study [29]. However, the biological effect of CPP-PNA complexes is limited as they are very significantly trapped in endosomes. Recent data indicate that conjugation of a lipid domain (a fatty acid) to CPP increases cellular bioavailability of the corresponding PNA [30,31]. Thus, such PNA-CatLip conjugates appear as pertinent anti-HBV agents. Interestingly, we have recently showed that CatLip peptides alone, in the absence of their PNA cargo, were able to inhibit viral replication [32].

Because chronic HBV-carriers have weak and unpaired virus-specific immune responses, the restoration of these responses via immunotherapeutic approaches such as DNA-based vaccines appears of particular interest for virus clearance. Thus, DNA vaccines targeting HBV proteins (envelope, core) are safe and able to induce potent humoral and cellular immune responses as demonstrated in different preclinical studies [33]. However, the first clinical trials showed only moderate efficacy of DNA vaccines in chronic HBV patients indicating a need for vaccine improvement. Different approaches to optimize DNA vaccine have shown that molecular adjuvants may considerably increase HBV vaccine potency [34,35].

HBV is the prototype member of the *Hepadnaviridae* family that includes mammalian and avian hepadnaviruses. One peculiar feature of these small, hepatotropic viruses is their common replication strategy, which has been extensively studied [36]. HBV genome consists of a partially double-stranded 3.2 kb DNA molecule termed relaxed-circular DNA (RC-DNA). Although HBV is a DNA virus, its replication involves reverse transcription of pregenomic (pg) RNA Upon hepatocyte infection, the nucleocapsids reach the nuclear pore, followed by the release of RC DNA into nucleoplasm where it is converted into cccDNA. This cccDNA, behaving as an episomal minichromosome, is a template for pgRNA synthesis and it plays a key role in the persistence of HBV infection. The pgRNA is selectively encapsidated in viral capsid via interactions with viral reverse transcriptase, which binds to its stem-loop structure called ε (epsilon) (encapsidaion signal). The reverse transcription of pgRNA subsequently leads to the initiation of first strand RC-DNA synthesis. The mature capsid can be enveloped with HBV envelope proteins in the endoplasmic reticulum (ER) and secreted into the blood. Alternatively, it can also re-enter the nuclei via intracellular recycling to replenish the cccDNA pool.

Due to the extremely narrow host range of HBV that infects only humans and chimpanzee, the closely related duck HBV (DHBV) provides a particularly useful model for evaluation of novel antiviral strategies. Indeed, this model offers not only an unique acellular system of enzymatically active hepadnaviral polymerase expression in a rabbit reticulocyte lysate, but it also allows to test the impact of novel antiviral strategies *in vitro*, in primary duck hepatocyte cultures (PDHs) and *in vivo*, in ducklings [37]. Although HBV cannot infect mice, mice harboring HBV transgene have been extensively used to study the immunopathogenesis of infection and to assess the therapeutic benefit of different antiviral strategies.

This review focuses on the use of CPPs and cationic polymers-based technologies for design of novel therapeutic approaches against chronic hepatitis B. The recent advances in the use of CPPs for cellular delivery of bioactive cargoes such as PNAs are presented. In addition, the results that allowed uncovering the ability of a modified CPP (CatLip) alone to drastically inhibit hepadnaviral morphogenesis are highlighted. Moreover, the benefit of HBV DNA vaccine conjugation to Man-CS-Phe for hepatitis B immunotherapy is detailed. Other systems for genes delivery are also described. Because current NUCs-based antiviral treatments cannot eliminate viral cccDNA from infected hepatocytes and lead to the selection of resistant mutants, alternative approaches such as antisense PNAs or DNA vaccines, targeting different steps of HBV replication cycle, appear of particular value for chronic hepatitis B therapy. 

## 2. Effect of CPP-PNA Conjugates on DHBV Replication

The development of novel therapeutic agents able to eradicate viral replication should be the challenge of future work in the management of chronic hepatitis B. Because the encapsidation signal ε plays a key role in the initiation of HBV reverse transcription [36,38], we have investigated the ability of an antisense PNA targeting this ε to inhibit viral replication in the DHBV infection model. We have previously demonstrated in cell-free *in vitro* system that such PNA targeting the DHBV ε can efficiently inhibit viral reverse transcription in a sequence-specific manner [28].

Because the main problem in the use of PNA as antiviral agents is their poor transport across the cell membrane, we first evaluated, the bioavailability of PNA alone or coupled to a CPP (CPP-PNA conjugate) *in vivo*, in the duck model. Thus fluorescein-labeled PNA targeting DHBV ε, which was conjugated to d-oligoarginine (FITC-PNA-CPP) was administrated into ducklings and the fluorescence was monitored in the cells. We have detected the presence of hepatocytes-associated fluorescence, indicating that this FITC-PNA-CPP was able to reach the duck liver [29].

Next, this PNA coupled to a CPP d-oligoarginine was tested for its ability to inhibit hepadnavirus replication *in vivo* in the DHBV infection model. To this end, ducklings were experimentally infected with DHBV inoculum and randomly assigned into different treatment groups that received antisense CPP-PNA therapy [29]. The mismatched PNA (MM-PNA) coupled to the same CPP d-oligoarginine or normal saline were administrated to control groups. The delivery of CPP-PNA conjugates led to delayed and reduced viremia in treated ducklings as compared with the untreated controls. Surprisingly, the injection of the CPP-MM-PNA conjugate, used as an additional control, also led to a decrease and delay in viremia in treated animals. In addition, liver DNA analysis indicated that both CPP-PNA conjugate and its corresponding CPP-MM-PNA, decreased intrahepatic viral DNA levels as compared with untreated controls. Because CPP-MM-PNA conjugate led also to the decrease in viremia and intrahepatic viral DNA, we hypothesized that this CPP (d-oligoarginine) alone may exhibit an antiviral activity. To further explore this hypothesis DHBV-infected ducklings were treated with different concentration of this CPP. Interestingly, a dose dependent decrease in viral replication was observed in CPP-treated ducklings as compared with untreated controls. Thus d-oligoarginine alone, in the absence of its PNA cargo, was able to inhibit DHBV replication [29].

**Figure 1:**
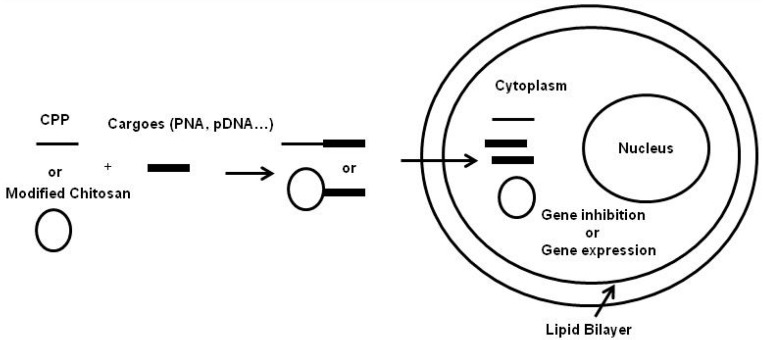
Nonviral gene delivery systems based on CPPs or modified chitosan (CS).

To improve PNA-based strategy for anti-HBV therapy, we have tested oligolysine for facilitating intracellular PNA delivery [29]. Treatment of DHBV-infected primary duck hepatocyte cultures (PDH) with anti-ε PNA coupled to oligolysine inhibited viral replication in a sequence-specific manner. Thus, neither CPP-MM-PNA nor oligolysine alone had a significant impact on DHBV replication. Collectively, these results indicate that conjugation of antisense PNA to CPPs allows successful intracellular delivery of PNA (Figure 1), which was able to inhibit hepadnaviral replication. Importantly, the antiviral activity of some CPPs may interfere with antisense specificity of CPP-PNA conjugates. Thus, our results suggest that the choice of CPPs used as vehicle for intracellular delivery of PNAs may play a critical role in the antiviral specificity of their cargos [29].

## 3. Modified CPPs (CatLip) as Novel Anti-HBV Agents

CPPs have broad biological activity, although their antibacterial potential has been more intensively studied than antiviral activity [39]. The antiviral activities of CPPs have been related to the interference with both viral adsorption and entry process of different enveloped viruses such as Herpes Simplex Virus (HSV), Vesicular stomatitis Virus (VSV) and Human Immunodeficiency Virus (HIV) [39,40]. In this regard, increasing evidences indicate that CatLip, resulting from chemical conjugation of CPPs to a lipid domain (such as fatty acids), have improved cellular uptake and endosomal escape [30,31]. Our recent discovery of anti-DHBV activity exhibited by oligoarginine, encouraged us to evaluate different CatLip for their ability to inhibit viral replication *in vitro*, in primary duck hepatocytes cells (PDHs) and in stably DHBV-transfected chicken hepatoma cells (LMH-D2) [32].

Thus, we performed screening of twelve oligoarginine-based CatLip, differing by fatty domains and oligoarginine lengths, which were tested for their capacity to interfere with DHBV replication. All tested CatLip inhibited DHBV replication without noticeable toxicity. Interestingly, Decanoyl-(DArg)_8_ peptide was identified as the most effective, since it induced a potent and a dose-dependent decrease of DHBV secretion in virus-infected PDHs or LMH-D2 cells as compared with the untreated controls. Such drastic decrease in viral release was not related to an eventual peptide toxicity as confirmed by MTT assay.

The specificity of Decanoyl-(DArg)_8_ antiviral activity was further studied by exploring the inhibition of viral secretion by (DArg)_8_ or decanoic acid [32]. Interestingly, decanoic acid alone was unable to decrease DHBV release, while (DArg)_8_ led to less marked inhibition (60%) as compared with Decanoyl-(DArg)_8_ treatment (90%). Additional analysis confirmed that Decanoyl-(DArg)_8_ induced a drastic decrease in viral release in both PDHs and LMH-D2 cells in dose-dependent manner. Thus, at 2 µM concentration, Decanoyl-(DArg)_8_ peptide reduced DHBV release by 90% at the end of seven days of treatment. This inhibition was comparable with that of Lamivudine at 100 µM. Moreover, the analysis of cellular uptake revealed that, at the same concentration, Decanoyl-(DArg)_8_-FITC was internalized more efficiently than (DArg)_8_-FITC in PDH cells. This finding supports a higher potent antiviral potential of Decanoyl-(DArg)_8_ as compared with (DArg)_8_.

In addition, our results showed that Decanoyl-(DArg)_8_ peptide does not inactivate the DHBV and does not interact with its receptor, because the pre-incubation of DHBV-positive inoculums and PDH cells with Decanoyl-(DArg)_8_ does not reduce DHBV infectivity [32]. Thus this CatLip peptide had no impact on the outcome of DHBV infection and on cells susceptibility to viral infection. In addition, these finding support that Decanoyl-(DArg)_8_ peptide is not a ligand of the recently identified hepadnaviral receptor [41,42].

Next, we investigated the impact of Decanoyl-(DArg)_8_ peptide treatment on DHBV replication by using PDH and LMH-D2 cells. Our findings showed that this treatment does not significantly affect viral transcription since viral RNA levels were unchanged. However, CatLip peptide treatment, promotes the accumulation of different viral DNA replicative forms. Furthermore, Decanoyl-(DArg)_8_ peptide treatment does not change intracellular cccDNA synthesis. In addition, in both PDH and LMH-D2 cells, no antiviral effect of CatLip peptide was observed on viral structural proteins (envelop and core) expression level [32].

To investigate the mechanism of Decanoyl-(DArg)_8_ action we explored its impact on intracellular distribution of different DHBV proteins and viral morphogenesis. Our results indicate that treatment with Decanoyl-(DArg)_8_ does not affect core proteins localization. Interestingly, viral envelope proteins accumulated in large clusters as shown by confocal laser scanning microscopy [32]. In addition, viral release from LMH-D2 cells measured by iodixanol-based gradient analysis showed that Decanoyl-(DArg)_8_ treatment drastically inhibited the release of both subviral particles and complete virions. By contrast naked viral capsids were still released, indicating that Decanoyl-(DArg)_8_ interferes with capsids envelopment [32]. Because the CPPs directly interact with biological membranes [43] these findings suggest that Decanoyl-(DArg)_8_ via its interaction with cellular membranes may induce drastic structural changes in viral envelope proteins, which may inhibit their association with the capsid and consequently block virus morphogenesis and secretion.

Taken together, our results provide the first evidence that Decanoyl-(DArg)_8_, alone, in the absence of its antiviral cargo, was able to interfere with late stages of hepadnavirus replication by drastic inhibition of viral egress [32]. Antiviral activity of CPPs has been described for different viruses, although their mode of action relies either on innate response activation (HSV, HIV) or inhibition of viral uptake (HSV, HIV, HCMV) [40]. Thus, this is also the first report demonstrating a new antiviral mechanism of CPPs action [32].

Our preliminary results suggest that Decanoyl-(DArg)_8_ is also able to inhibit, in a dose-dependent manner, human HBV morphogenesis. This is of particular interest, since in contrast to current antiviral drugs that inhibit HBV polymerase leading to resistant variant emergence, Decanoyl-(DArg)_8_, by targeting another step of viral life cycle, may represent a novel class of therapeutics for chronic hepatitis B treatment. In addition due to the high immunogenicity of core antigen, the secretion of large amounts of naked nucleocapsids generated via Decanoyl-(DArg)_8_ treatment may be valuable for immune responses stimulation in HBV patients. Different CPPs and their biological activities are summarized in Table 1.

**Table 1:** Different CPPs and their therapeutic potential against hepatitis B.

CPPs	Cargo	Cells/Animal Models	DHBV/HBV Infection	Bilogical Activities
(DArg)_8_	PNA	PDHs, Peking Ducklings	DHBV	Inhibition of DHBV replication [29]
Decanoyl-(DArg)_8_	-	PDHs, LMH-D2, HepG2.2.15	DHBV/HBV	Drastic Inhibition of late stages of DHBV replication Inhibition of HBV release [32]

## 4. Benefit of Chitosan-Mediated Gene Uptake

The CPPs also considerably improve cellular uptake of natural cationic polymers such as chitosan (CS). Indeed, CS appears as an interesting nonviral vector for genes delivery due to its low cytotoxicity and remarkable biocompatibility. However the poor cellular uptake of unmodified CS limited its clinical applications. In this regard, recent data indicate that linoleic acid and penetratin CPP dual-functionalized chitosan (CS-Lin-Pen) constitute the polymeric micelles which promote efficient pDNA condensation and improve its stability [44]. Moreover, pDNA polyplexes transfection experiments showed that in Human embryonic kidney (HEK 293), in Chinese hamster ovary (CHO) and in human cervical adenocarcinoma (HeLa) cells, CS-Lin-Pen micelles exhibited ~34–40-fold higher transfection of pDNA in comparison with unmodified chitosan. Thus, these results indicate that CS-Lin-Pen micelles could efficiently transport pDNA into several cell lines (Figure 1). Altogether, these findings strongly suggest that chitosan-based cationic micelles appear as an efficient nonviral gene carrier that, importantly, showed no toxicity [44] (Table 2).

**Table 2:** Other system for gene delivery.

Gene Delivery System	Cationic Polymers	Cargo	Target	Bilogical Activities	Toxicity
CPP-modified chitosan	-CS-Lin-Pen	pDNA	HEK293, CHO, HeLa cells	Efficient protection of pDNA from DNAse I attack. Facilitates the celluar uptake of the pDNA to the nucleus for the transcription [44].	-
	-LTVSPWY-PEG-CS	magnetic nanoparticles	A549 cells, SKOV-3 cells	Promote the cellular targeting the MNPs [45].	
Mannose or Hydrophobic-modified Chitosan (CS)	-Man-CS-Phe	pDNA	RAW264.7, DC2.4 cells, Balb/C mice	Target of APC induction of humoral and cellular HBV-specific immune response [35].	-
	-Caproic acid-grafted chitosan (CGC-15-polymers)	pDNA	HEK293 cells	Increase pDNA, cell uptake and gene expression [46].	-
Cationic nanoparticles	-Poly(dl-Lactide*-co*-glycolide) (PLGA) -Poly(dl-Lactic acid) (PLA)	pDNA	HEK293 cells	Enhance pDNA expression by its prolonged release into cells [47].	-

In another study, Chopra had studied the heptapeptide LTVSPWY, having a high binding specificity for human mammary adecarcinoma SKBR3 cells, which was first linked to poly (ethyleneglycol) and thereafter conjugated to chitosan (Table 2). Interestingly, the LTVSPWY-PEG-CS complexes were able to promote cellular targeting of magnetic nanoparticles (MNPs) in both A549 and SKOV-3 cells [45].

Several other CS-based systems for genes delivery and their biological activities are summarized in Table 2.

## 5. Modified Chitosan-HBV DNA Vaccine Conjugates Improve Cellular and Humoral Responses

Individuals with chronic hepatitis B infection have very weak and unpaired HBV-specific cellular and humoral responses. Thus, the immunotherapeutic approaches leading to the stimulation and restoration of these responses in chronic HBV-carriers appears as essential for resolution of infection [48]. In this regard, DNA vaccines represent a pertinent alternative therapeutic strategy for chronic hepatitis B treatment [33,49]. Indeed, HBV surface antigen (HBsAg) that is particularly immunogenic can be used as candidate molecule in this DNA vaccination technology. To improve DNA vaccine delivery and thus the presentation of the encoded antigen by professional antigen presenting cells (APCs) (dendritic cells and macrophages), nonviral vectors have been extensively studied [50,51]. Moreover, because the chitosan backbone can be easily modifiable by chemical groups, it represents an attractive tool for plasmid DNA (pDNA) release into the cells.

The first limitation of unmodified CS as delivery system of pDNA is related to their poor cellular internalization and partial dissociation of pDNA [52]. However, a recent study has established that the coupling of chitosan backbone to hydrophobic molecules reduce its net positive charge in order to promote its intracellular dissociation of the polyplexes, thus leading to pDNA liberation into the cells [53] (Figure 1).

The second problem of CS-based delivery systems is their weak cell specificity. To increase their specificity toward APC, the chitosan was coupled to mannose because APC overexpress mannose receptor (MR) on their surface and such a coupling could improve the cellular internalization via MR-mediated endocytosis mechanism [54]. Thus, both approaches would improve DNA vaccine uptake and efficiency.

In the recent report both approaches described above were combined [35]. Thus, the CS backbone was modified with hydrophobic amino acid l-phenylalanine in order to improve their APC uptake and also with a-d-mannopyranosylphenyl isothiocyanate to enhance the APC specificity [35]. The benefit of different coupling ligands such as chitosan-phenylalanine (CS-Phe), chitosan-phenylalanine-mannose (Man-CS-Phe) was analyzed *in vitro* in Murine macrophage RAW 264.7 and mouse dendritic cells DC 2.4 cells and the most efficient was selected and used for the delivery of HBsAg encoding pDNA *in vivo*, into Balb/c mice [35] (Table 2).

Firstly, the Man-CS-Phe copolymer was synthetized and characterized, followed by condensation of cationic micelles (CM) with pDNA leading to polymer/pDNA polyplexes. These polyplexes have been tightly condensed by protonated amines via electrostatic interaction representing approximately 90% at the weight ratio of 40. In a previous study, Bao *et al.* [55] reported that the great condensation of pDNA into the nanoscale polyplexes confered them an efficient protection against the cellular nucleases. Thus, the ability of these tightly condensed pDNA polyplexes to resist to cellular nuclear digestion was analyzed. The results showed that the naked pDNA was totally degraded by the DNase I while the cationic micelle/pDNA polyplexes remained efficiency protected [35]. The cytotoxicity study indicated that the cationic micelles and their corresponding polyplexes did not exhibit toxicity in the RAM 264.7 and DC 2.4 cells at tested concentrations [35].

Next, cellular uptake analysis of different polyplexes in RAM 264.7 mouse macrophages and DC 2.4 mouse dendritic cells, which express abundant MR on their surface [54], revealed that Man-CS-Phe induced the best pDNA cellular internalization followed by CS-Phe, whereas CS led to only a very low pDNA cellular uptake. Because Man-CS-Phe polymers contain the mannose, the pre-incubation of cells with this sugar considerably decreased cellular internalization of the Man-CS-Phe/pDNA polyplexes due to a competition mechanism between mannose alone and Man-CS-Phe/pDNA polyplexes at the cellular mannose receptor [35]. These results suggest that the Man-CS-Phe/pDNA polyplexes were internalized into the cells via the mannose receptor-mediated endocytosis mechanism [56], which improves Man-CS-Phe/pDNA polyplexes uptake compared to that of CS-Phe/pDNA polyplexes.

Polyplex transfection experiments confirmed the results described above. Indeed, in RAW 264.7 cells, the Man-CS-Phe/ Plasmid DNA encoding green fluorescence protein (pGFP) polyplexes showed better transfection, which was 20-fold superior to CS/pGFP polyplexes and 1.5-fold superior to that of CS-Phe/pGFP polyplexes. Furthermore, the pGFP transfection efficacy of Man-CS-Phe polymer was significantly (*p* ˂ 0.05) higher than FuGENE HD. In addition, competition experiment with mannose demonstrated the key role played by MR in the intracellular uptake of Man-CS-Phe/gene polyplexes. Finally, the gene transfection efficiency in RAW 264.7 cells was higher than that in DC 2.4 cells [35].

Based on these encouraging results, the humoral anti-HBsAg response was investigated in mice. The results showed that immunization of mice with different vaccine formulation such as CS-Phe/pHBsAg and Man-CS-Phe/pHBsAg polyplexes induced a significantly higher (*p* ˂ 0.05) level of anti-HBsAg-specific IgG antibody responses in comparison with PBS control group [35]. In addition, Man-CS-Phe/pHBsAg polyplexes exhibited the strongest HBsAg-specific antibody responses compared to that obtained with CS-Phe/pHBsAg polyplexes, suggesting that the Man-CS-Phe/pHBsAg polyplexes efficiently targeted the dendric cells and were able to stimulate potent humoral and cellular immune responses [57]. Thus, both polyplexes led to a better HBsAg-specific antibody response than naked pHBsAg due to the greater stability and cellular internalization of the condensed pHBsAg, resulting in higher gene expression. [35]. Taken together, these results provide the first evidence that Man-CS-Phe cationic micelles could represent a powerful tool in DNA vaccine-based approach to enhance HBV-specific humoral immune responses in chronic virus-carriers [35].

The complete resolution of chronic hepatitis B infection needs an efficient humoral response in combination with a potent virus-specific cellular immunity [58]. Thus, the cellular immunity against HBV was investigated through the lymphocyte proliferation analysis and measuring of the cytokines (IFN-γ and IL-4) expression synthesized by splenocytes of immunized mice [35]. The results showed that, the immunization of mice with Man-CS-Phe/pHBsAg polyplexes led to significantly higher (*p* ˂ 0.05) cellular immune response than all other DNA vaccine formulations (CS-Phe, FuGENE HD) which result in a large lymphocyte proliferation compared to PBS control and naked pHBsAg groups [35]. Such strong lymphocyte proliferation reflects a potent stimulation of antigen-specific CD4^+^ helper T-cells [59]. These cellular immune responses generated by the different vaccine formulations were in accordance with previously obtained anti-HBsAg-specific IgG antibody responses previously obtained.

Similarly, the analysis of cytokine expression (IFN-γ and IL-4) by immunized mice showed that Man-CS-Phe/pHBsAg polyplexes induced a significantly (*p* ˂ 0.05) higher IFN-γ response than all other immunized groups, while the IL-4 expression level remained unchanged [35]. In this regard, IFN-γ plays an important role in the stimulation of several immune actors and increase of some immune molecule expression such as MHC classes I and II [60]. Pioneering study in the chimpanzee model showed a key role of IFN-γ expression in the liver during resolution of HBV infection [61]. Moreover in the DHBV model we have previously demonstrated the significant role of duck IFN-γ (DuIFN-γ) in the resolution of viral infection [62]. In this context the results of Layek *et al.* [35] suggest that immunization with Man-CS-Phe/pHBsAg can strongly stimulate Th1 responses, which are of particular importance for the control of HBV infection. In addition, the *in vivo* toxicity study did not reveal a significantly cytotoxicity within immunized mice.

Altogether, these results convincingly demonstrated that mannosylated phenylalanine grafted chitosan (Man-CS-Phe), a nonviral vector for DNA vaccination, can exclusively target APCs and induced potent humoral and cellular HBV-specific immune responses in mice model (Table 2). Finally, these data provide the first illustration [35] that hepatitis B DNA vaccine/Man-CS-Phe can represent a promising tool for effective and targeted delivery of DNA vaccine to APCs that could be particularly useful for therapy of chronic HBV-carriers.

Because, unmodified chitosan is less effective as vehicle, another chemical modification was performed involving the caproic acid with three substitution degrees. In this study, the effect of the degree of substitutions of caproic acid on pDNA binding, cellular uptake and transfection efficiency was investigated in HEK 293 and HeLa cells [46]. The results showed that the caproic acid-grafted chitosan (CGC) enhanced pDNA stability. Interestingly, the three substitution degrees (CGC-5, CGC-15 and CGC-25) improved cellular uptake of pDNA [46]. In addition, the CGC-15 polymer exhibited 31-fold higher gene expression compared to unmodified CS. Taken together, this hydrophobic modification of chitosan enhances cellular internalization of pDNA and consequently its expression. Thus caproic acid-grafted chitosan appears as a promising nonviral gene delivery vector [46].

In another study, the cationic nanoparticles as Poly(dl-Lactide-co-glycolide) termed PLGA and Poly(dl-Lactic acid termed PLA were used to release pDNA into the HEK 293 cells [47]. The results demonstrated that PLGA and PLA cationic nanoparticles increased pDNA cellular uptake in prolonged manner and promote its intracellular expression [47].

## 6. Conclusions

Taken together, these encouraging results generated in preclinical studies conducted in animal models (duck and mouse) point out the usefulness of CPPs for the development of novel antiviral strategies to fight hepatitis B infection.

In addition, by conjugation to cationic polymers such as chitosan, the CPP improved cellular uptake of pDNA and its intracellular expression. Thus, CPP-modified CS was more effective than unmodified CS and considerably enhanced gene delivery. The cationic nanoparticles are likewise used as vehicle to transfer biological molecules into the cells. These different gene delivery systems appear as promising nonviral strategies, which could represent a potential alternative for chronic hepatitis B therapy. Thus, it will be of particular interest to evaluate the ability of caproic acid-grafted CS or CS-Lin-Pen micelles to enhance therapeutic potential of DNA vaccine-based immunotherapy against chronic HBV infection.

With the continued development of such innovative, nonviral gene carriers, better tools for antisense PNAs and DNA vaccine delivery await to be tested in the most relevant experimental models of HBV infection. Because the increasing evidence indicates the importance of combination therapies to fight chronic hepatitis B, the association of such novel, nonviral gene carriers with the current nucleoside analogues-based antiviral strategies, appears of particular interest for design of potent anti-HBV therapeutics.
